# Urinary lithogenic factors in urolithiasis patients with kidney disease and correlation with renal function

**DOI:** 10.3389/fmed.2026.1826969

**Published:** 2026-08-03

**Authors:** Qian Dong, Huan Xu, Pengjie Xu, Jiang Liu

**Affiliations:** Department of Nephrology, The Affiliated Lihuili Hospital of Ningbo University, Ningbo, China

**Keywords:** citrate/creatinine ratio, kidney disease, kidney stones, lithogenic factors, renal function

## Abstract

**Background:**

Patients with kidney disease have a high incidence of kidney stones, yet the specific mechanisms remain elusive. The purpose of this study was to explore the connection between urinary lithogenic factors and renal function.

**Methods:**

We retrospectively analyzed 150 patients with kidney disease -associated nephrolithiasis and 84 stone-forming patients with common nephrolithiasis. The renal disease group comprised 69 renal transplant recipients, 36 patients with CKD stages 1–2, and 45 patients with CKD stages 3–4. Urinary lithogenic factors and renal function indicators were compared and correlated.

**Results:**

Patients with kidney disease showed significantly higher calcium/creatinine and magnesium/creatinine ratios, and lower citrate/creatinine ratios and urinary uric acid levels, compared to those with common nephrolithiasis (all *P* < 0.05). No significant differences were observed in oxalate/creatinine or phosphate/creatinine ratios. Multivariate analysis identified Scr, BUN, PCR, eGFR, calcium/creatinine, citrate/creatinine, magnesium/creatinine, and urinary uric acid as independent factors associated with renal disease-associated nephrolithiasis (all *P* < 0.05). The distribution of core lithogenic factors differed across renal disease subtypes: citrate/creatinine was the core factor in renal transplant recipients, whereas calcium/creatinine was the core factor in patients with CKD stages 1–2, with urinary uric acid as a secondary factor. The prevalence of hypocitraturia, defined as a urinary citrate-to-creatinine ratio < 0.22 mmol/mmol, was markedly elevated across all renal disease subgroups (93.5% in transplant recipients, 100% in CKD stages 3–4, and 88.9% in CKD stages 1–2) compared with 66.7% in the general nephrolithiasis group. Correlation analysis revealed that declining renal function was positively associated with calcium/creatinine and magnesium/creatinine ratios, and negatively associated with citrate/creatinine ratio (all *P* < 0.05).

**Conclusion:**

Patients with kidney disease had increased urinary calcium and magnesium excretion and decreased citrate levels compared to people with common nephrolithiasis. Kidney disease is associated with distinct alterations in urinary lithogenic factors, particularly hypocitraturia, with subtype-specific patterns that may guide targeted prevention strategies. These findings were based on a cross-sectional assessment using a single first-morning urine sample adjusted for urinary creatinine and should be validated with 24-h urine metabolic evaluation in future studies.

## 1 Introduction

Patients with kidney disease frequently experience disturbances in mineral metabolism, acid–base imbalance, and abnormalities in uric acid (UA) excretion, all of which significantly increase the risk of urinary stone formation. Kidney disease is one of the most common conditions in urological surgery. The primary clinical manifestations include flank pain, hematuria, frequent urination, and urgency. If left untreated, progression may result in recurrent urinary tract infections, acute urinary obstruction, and in severe cases, acute or chronic renal failure or even nephrectomy (1–3). Epidemiological data indicate that kidney disease is characterized by a high recurrence rate, with approximately 50% of patients experiencing recurrence within 10 years and about 75% within 20 years (4). Therefore, early identification of kidney disease is crucial for reducing the recurrence rate of kidney stones.

Renal calculi are primarily composed of crystalline substances and can be classified into five major types according to their chemical composition: calcium oxalate stones, calcium phosphate stones, struvite (magnesium ammonium phosphate hexahydrate) stones, UA stones, and cystine stones (5, 6). There are distinct differences among these types of calculi in terms of their formation mechanisms and physicochemical characteristics (7, 8). Currently, chemical analysis and infrared spectroscopic analysis are the commonly used methods for determining the composition of calculi (9). However, these techniques require the stones to be expelled beforehand or surgically removed, which limits their application in guiding early treatment strategies (9, 10). Therefore, the identification of reliable *in vivo* methods for determining stone composition is of critical importance.

With the advancement of research, clinical evidence has indicated that, compared with the general population, patients with kidney disease exhibit a higher incidence of urinary calculi. Owing to renal dysfunction–associated disturbances in mineral metabolism, acid–base imbalance, and reduced UA excretion, these patients often present with distinct stone composition profiles (11–13). Urinary lithogenic factors refer to the concentrations and ratios of various stone-promoting and stone-inhibiting substances in urine that are closely associated with stone formation, mainly including calcium ions (Ca^2^?), oxalate ions (C_2_O4^2^?), citrate ions (C6H5O7^3^?), and magnesium ions (Mg^2^?). Urinary lithogenic factor analysis is a non-invasive method for evaluating the concentrations and relative proportions of stone-promoting and stone-inhibiting substances in urine (14). Urinary stone formation factor analysis is a non-invasive method used to assess the concentrations and relative proportions of lithogenic and inhibitory substances in urine (15). In view of the current knowledge gap regarding stone formation characteristics in patients with kidney disease, the present study systematically analyzed the urinary stone formation factor profiles in patients with kidney disease–associated nephrolithiasis and explored their correlations with renal function parameters. These findings are expected to directly inform individualized therapeutic strategies, facilitate targeted correction of metabolic abnormalities, and ultimately refine preventive approaches in clinical practice.

## 2 Methods

### 2.1 Research participants

**Figure 1:**
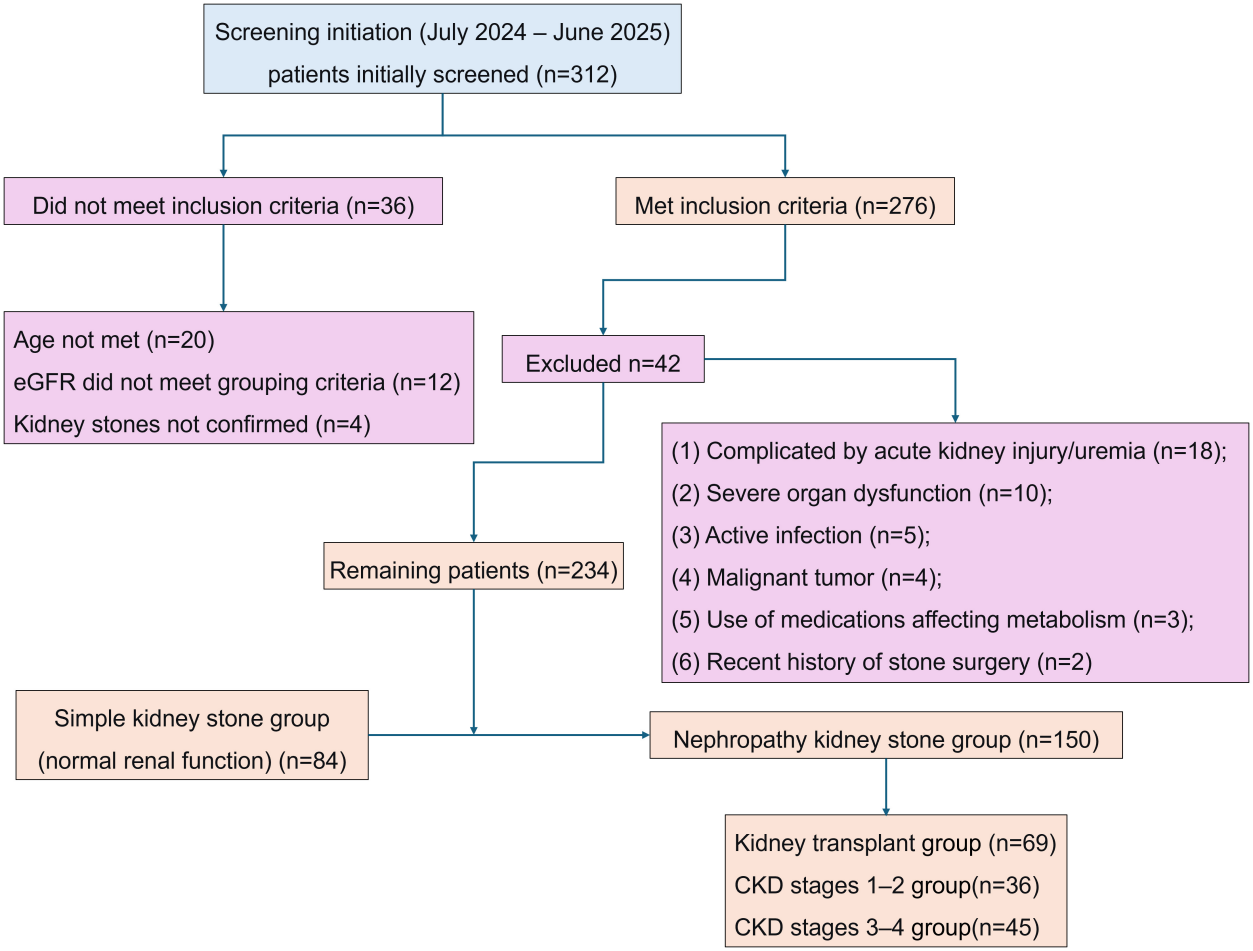
Flowchart.

This was a single-center retrospective observational study. 150 patients with kidney disease–associated nephrolithiasis who were admitted to our hospital between July 2024 and June 2025 were enrolled, including 69 renal transplant recipients, 36 patients with CKD stages 1–2, and 45 patients with CKD stages 3–4. In addition, 84 stone-forming patients with common nephrolithiasis during the same period were included as controls. According to guideline-based criteria, the 150 patients with kidney disease–associated nephrolithiasis were stratified into three groups: the CKD stages 1–2 group (*n* = 36), the CKD stages 3–4 group (*n* = 45), and the renal transplant group (*n* = 69). The included patients with CKD had a relatively long disease duration and varying degrees of renal impairment, with glomerular diseases constituting the predominant underlying renal disorders. The specific distribution was as follows: 76 patients had primary glomerular diseases, including 31 with membranous nephropathy, 25 with IgA nephropathy, and 20 with minimal change disease; 74 patients had secondary glomerular diseases, including 38 with diabetic kidney disease, 22 with hypertensive nephropathy, and 14 with obesity-related kidney disease. Ethical approval for the study was granted by our hospital ethics committee (KY2025SL148-01), and all procedures were conducted in accordance with the ethical standards of the 1964 *Declaration of Helsinki* and its subsequent amendments. Informed consents were obtained from patients. Details are shown in Figure 1.

### 2.2 Inclusion, exclusion, and elimination criteria

Inclusion criteria: (1) Age between 18 and 85 years; (2) Patients with kidney disease–associated nephrolithiasis included those with CKD (stages 1–4) and renal transplant recipients. According to the KDIGO 2024 Clinical Practice Guideline, CKD was defined as a chronic condition characterized by abnormalities of kidney structure or function persisting for more than 3 months and having implications for health. The specific diagnostic criteria included: (a) markers of kidney damage, such as albuminuria [urinary albumin-to-creatinine ratio ≥30 mg/g], urinary sediment abnormalities, tubular disorders, histological abnormalities, or structural abnormalities detected by imaging; and (b) a glomerular filtration rate (eGFR) < 60 mL/min/1.73 m^2^. CKD was diagnosed when either criterion (a) or (b) persisted for more than 3 months. Accordingly, patients with CKD stages 1–2 in this study were diagnosed on the basis of markers of kidney damage, such as persistent microalbuminuria, haematuria, or urinary casts, whereas patients with CKD stages 3–4 also met the criterion of an eGFR < 60 mL/min/1.73 m^2^; (3) kidney stones were confirmed by imaging (CT or KUB ultrasound).

Exclusion criteria: (1) Patients with concomitant acute kidney injury, uremia, hereditary kidney diseases, or urinary tract malformations were excluded; (2) patients with severe dysfunction of the heart, liver, or kidneys; (3) patients with active systemic infections; (4) patients with malignant tumors; (5) patients with severe neurological disorders or cognitive impairments; (6) pregnant or lactating women. (7) Patients currently taking medications that affect stone metabolism (such as thiazide diuretics, allopurinol, or potassium citrate); (8) Patients with a history of stone surgery or extracorporeal shock wave lithotripsy within the past 3 months. In addition, to minimize the impact of disease status on urinary sample stability, all urine samples were collected during the non-acute phase and in the absence of therapeutic interventions that could potentially alter urinary composition.

Elimination criteria: patients whose data were incomplete or insufficient for evaluation, thereby potentially affecting statistical analysis, were excluded. To minimize the potential confounding effects of specific disease conditions on stone formation, this study further excluded patients with clearly defined metabolic disorders that can independently promote stone formation, including complex urinary tract obstruction, distal renal tubular acidosis, and hyperoxaluria, thereby allowing the analysis to focus on changes in lithogenic factors associated with CKD itself. In addition, to systematically control for the potential effects of acid–base balance on lithogenic factors, morning urinary pH and serum bicarbonate (HCO3?) were routinely measured in all patients at enrolment. The results showed that urinary pH ranged from 5.5 to 6.5 in all included patients, which was within the normal physiological range, while serum bicarbonate levels were within the normal reference range of 22–28 mmol/L. Therefore, the cohort as a whole was considered to be in acid–base balance. These acid–base parameters were not included in the primary statistical analyses, but their potential confounding effects on the study conclusions were effectively controlled.

### 2.3 General information

As a retrospective study, all clinical data were obtained from hospital medical record system. Collected variables included sex, age, history of comorbidities (diabetes diagnosed according to the 2019 diabetes management guidelines jointly developed by the European Society of Cardiology and the European Association for the Study of Diabetes (16)); hypertension diagnosed in accordance with relevant standards in the ACC/AHA hypertension guidelines (17), disease duration (obtained by professional clinicians using structured communication techniques), smoking history (defined as smoking ≥100 cigarettes in the past year), alcohol consumption history (defined as alcohol intake at least once per week for five consecutive months), and BMI, systolic blood pressure and diastolic blood pressure.

### 2.4 Detection of urinary lithogenic factors and urinalysis

To minimize the potential confounding effects of disease status and therapeutic interventions on urinary composition, all participants were required to follow a standardized “low-oxalate/low-purine diet” guideline for at least 24 h before urine collection. This involved avoiding specific foods such as spinach, nuts, animal offal, and seafood to minimize short-term dietary fluctuations that could affect the urinary concentrations of lithogenic factors such as oxalate. Water intake was also strictly controlled. Urine samples were collected prior to stone passage events and during non-acute symptomatic periods (e.g., avoiding episodes of renal colic) to eliminate potential interference from acute attacks or therapeutic interventions on urinary or serum biomarkers. A 10-mL aliquot of freshly voided midstream morning urine was collected for analysis, placed in a sterile Eppendorf (EP) tube, immediately cooled in an ice bath, and delivered to the laboratory within 20 min. The samples underwent centrifugation (1000 × g, 15 min, 4 C), after which the supernatant was collected and submitted for analysis.

### 2.5 Determination of urinary creatinine and UA

Urinary creatinine (Scr) and UA levels were measured by laboratory technicians using the AU480 fully automated biochemical analyzer (Beckman Coulter, United States) and its corresponding reagents. Urinary creatinine was determined by the sarcosine oxidase method, while UA was measured using a colorimetric assay. The manufacturer's instructions were strictly adhered to during all procedures.

Urinary biochemical analysis: the detection of urinary anions and cations—including oxalate, citrate, phosphate, calcium, and magnesium—was conducted using ion chromatography. Anions such as oxalate, citrate, and phosphate were measured with ion chromatographs (models: LAB-ZP-YQ-010 and LAB-ZP-YQ-181), while cations such as magnesium and calcium were measured using ion chromatograph model LAB-ZP-YQ-011. The separation of coexisting anions and cations was achieved based on the principle of ion exchange. Due to differences in retention times on the chromatographic column, different substances were eluted by specific eluents at distinct times, enabling effective separation. The separated components were then quantitatively analyzed by measuring the corresponding conductivity signals in the conductivity cell, from which the concentrations of the respective substances were obtained. All procedures were carried out by laboratory personnel in strict compliance with the standardized operational protocols of the instruments. Since urinary solute concentrations are easily influenced by factors such as water intake and urine concentration, the creatinine correction method was adopted to minimize these interferences and improve the accuracy and comparability of the results. Each solute concentration was divided by the corresponding urinary creatinine concentration, and the ratios of calcium/creatinine, oxalate/creatinine, phosphate/creatinine, citrate/creatinine, and magnesium/creatinine were used as the final analytical indicators.

All ion chromatography analyses were quantified using the external standard method, and calibration curves were validated within the linear range (*R*^2^ > 0.99). The coefficient of variation (CV) for repeated sample measurements was < 5%. During data analysis, the creatinine normalization method was applied to control for the influence of urine concentration. Specifically, each ion concentration (mmol/L) was divided by the urinary creatinine concentration (mmol/L), and the results were expressed as mmol/mmol.

The risk thresholds and diagnostic criteria for each lithogenic factor were defined as follows: hypocitraturia was defined as a urinary citrate-to-creatinine ratio < 0.22 mmol/mmol, equivalent to a 24-h urinary citrate excretion < 320 mg/24 h; hypercalciuria was defined as a random urinary calcium-to-creatinine ratio >0.21 mmol/mmol; hyperoxaluria was defined as a 24-h urinary oxalate excretion >40 mg/24 h; hypomagnesuria was defined as a 24-h urinary magnesium excretion < 35 mg/24 h; and hyperuricosuria was defined as a 24-h urinary uric acid excretion >750 mg/24 h, with sex-specific thresholds of >600 mg/24 h for women and >800 mg/24 h for men. These thresholds were mainly based on the standards for 24-h urinary metabolic evaluation recommended by the International Society of Urology and the EAU/AUA guidelines, together with reference ranges reported in relevant domestic and international population studies.

Twenty-four-hour urine analysis is currently regarded as the gold standard for assessing the metabolic burden of lithogenic factors. In this study, lithogenic factors were measured using random first-morning urine samples and adjusted for urinary creatinine to reduce bias caused by variations in urine dilution and concentration. Although this method is widely used in epidemiological screening and is operationally convenient, it should be emphasized that it reflects metabolic status at only a single time point and cannot fully replace 24-h urinary metabolic analysis, the gold-standard method for comprehensively assessing metabolic excretion burden. Therefore, the metabolic evaluation performed in this study should be regarded as preliminary screening and exploratory correlation analysis. Future studies should incorporate complete 24-h urine data to improve the robustness of the conclusions.

### 2.6 Laboratory examinations

A 4 mL fasting peripheral venous blood sample was collected from each participant and placed into a vacuum blood collection tube. The samples were promptly transported to the laboratory, centrifuged at room temperature to separate serum and plasma, and subsequently stored at −80 C until analysis.

Hematological parameters: red blood cell count (RBC), white blood cell count (WBC), neutrophil count (NEU), and hemoglobin concentration were measured using an automated hematology analyzer.

Blood glucose and lipid profile: fasting blood glucose (FBG) and triglycerides (TG) were measured using an automated biochemical analyzer. Glycated hemoglobin (HbA1c) was determined by high-performance liquid chromatography (HPLC).

Liver function tests: serum alanine aminotransferase (ALT) and aspartate aminotransferase (AST) levels were measured using the rate method with corresponding reagent kits on an automated biochemical analyzer.

Renal function assessment: serum creatinine (Scr) was measured using an enzymatic creatinine assay kit on an automated biochemical analyzer. The eGFR was calculated using the CKD-EPI equation.

### 2.7 Statistical analysis

Statistical analyses were conducted using SPSS (version 26.0 or later). The normality of the data was assessed using the Kolmogorov-Smirnov test and visually inspected through Q-Q plots. Continuous variables following a normal distribution were expressed as mean±standard deviation, whereas non-normally distributed variables were presented as median (interquartile range). For normally distributed variables with equal variances, intergroup comparisons were performed using the independent-samples *t*-test. In contrast, for variables with unequal variances or non-normal distributions (e.g., age and Scr), the Mann-Whitney U test was utilized. Categorical variables were expressed as frequencies and percentages, and the chi-square (χ^2^) test was used to compare groups. The associations between renal function indicators and urinary lithogenic factors were evaluated using Spearman's rank correlation coefficient, and linear relationships were further explored through linear regression models. All statistical visualizations and regression analyses were conducted using GraphPad Prism (v8.0). To ensure the statistical power of the study, a *post hoc* power analysis was performed using GPower 3.1 based on the observed effect size (Cohen's *d* = 0.46) for the primary outcome (i.e., the difference in urinary citrate-to-creatinine ratio between the kidney disease group and the non-renal stone group), a predefined two-sided alpha level (α = 0.05), and the fixed sample sizes (kidney disease group *n* = 150; non-renal stone group *n* = 84). The analysis yielded a statistical power of 0.92, which exceeds the conventional threshold of 0.8, indicating that the present sample size provides adequate power to detect clinically meaningful between-group differences.

## 3 Results

### 3.1 Analysis of baseline characteristics between patients with kidney disease–associated nephrolithiasis and those with common nephrolithiasis

#### 3.1.1 General information

There were no statistically significances between patients with kidney disease complicated by nephrolithiasis and those with common nephrolithiasis in terms of sex distribution, mean age, mean BMI, proportion of smokers, proportion of drinkers, systolic blood pressure, diastolic blood pressure, prevalence of hypertension, prevalence of diabetes mellitus, RBC, WBC, NEU, hemoglobin concentration, FBG, TG, HbA1c, ALT, or AST levels (all *P* > 0.05, Table 1).

**Table 1:** Comparison of general characteristics between patients with kidney disease–associated nephrolithiasis and those with common nephrolithiasis.

Variable		Kidney disease and kidney stones (*n* = 150)	Common nephrolithiasis (*n* = 84)	*χ^2^*/*Z*/*t*	*P*
Gender (*n*, %)	Male	106 (70.67)	61 (72.62)	0.100	0.752
	Female	44 (29.33)	23 (27.38)		
Age [Years, M (P25, P75)]		51.00 (39.80, 61.00)	45.00 (40.30, 54.00)	−1.748	0.080
BMI (kg/m^2^)		23.27 ± 2.91	22.85 ± 1.63	1.219	0.224
Smoking history (*n*, %)		25 (16.67)	12 (14.29)	0.228	0.633
Alcohol history (*n*, %)		17 (11.33)	10 (11.90)	0.017	0.896
Systolic blood pressure (mmHg)		133.00 ± 18.09	132.50 ± 11.78	0.228	0.820
Diastolic blood pressure (mmHg)		79.76 ± 11.68	78.91 ± 8.56	0.585	0.559
Comorbidities (*n*, %)	Hypertension	34 (22.67)	17 (20.24)	0.186	0.667
	Diabetesmellitus	13 (8.67)	6 (7.14)	0.167	0.683
Red blood cell count ( × 10^1^^2^/L)		5.29 ± 1.07	5.41 ± 1.02	0.837	0.404
White blood cell count ( × 10^9^/L)		10.25 ± 2.33	9.87 ± 1.97	1.263	0.208
Neutrophil count ( × 10^9^/L)		7.05 ± 2.56	6.58 ± 1.85	1.480	0.140
Hemoglobin (g/L)		157.26 ± 18.03	155.09 ± 16.33	0.913	0.362
FBG (mmol/L)		4.15 ± 1.09	4.39 ± 0.98	1.674	0.095
TG (mmol/L)		1.23 ± 0.36	1.16 ± 0.31	1.498	0.136
HbA1c (%)		6.04 ± 1.17	5.79 ± 1.15	1.578	0.116
ALT (U/L)		22.67 ± 11.55	21.45 ± 7.82	0.846	0.398
AST (U/L)		20.51 ± 6.79	19.83 ± 5.91	0.769	0.443

### 3.2 Analysis of the association between renal function parameters and stone composition in patients with kidney disease–associated nephrolithiasis

#### 3.2.1 Comparison of renal function parameters between patients with kidney disease–associated nephrolithiasis and those with common nephrolithiasis

The eGFR was lower in the patients with kidney disease and kidney stones than patients with common nephrolithiasis, and the difference was statistically significant (*P* < 0.05). Scr, blood urea nitrogen (BUN), and protein-to-creatinine ratio (PCR) levels were significantly higher in patients with kidney disease–associated nephrolithiasis compared with those with common nephrolithiasis (all *P* < 0.05, Table 2).

**Table 2:** Comparison of renal function test results between patients with kidney disease–associated nephrolithiasis and those with common nephrolithiasis.

Variable	Kidney disease–associated nephrolithiasis (*n* = 150)	Common nephrolithiasis (*n* = 84)	*t* value	*P* value
Scr (μmol/L)	129.54 ± 83.53	61.36 ± 11.90	7.432	< 0.001
BUN (mmol/L)	8.96 ± 6.69	6.50 ± 1.45	3.324	0.001
PCR (mg/mg)	1.66 ± 1.14	0.51 ± 0.14	9.198	< 0.001
eGFR (mL/min/1.73 m^2^)	70.40 ± 25.35	106.53 ± 29.33	12.386	< 0.001

#### 3.2.2 Comparison of urinary lithogenic factor profiles between patients with kidney disease–associated nephrolithiasis and those with common nephrolithiasis

Patients with kidney disease–associated nephrolithiasis exhibited significantly higher calcium/creatinine and magnesium/creatinine ratios compared with those with common nephrolithiasis (all *P* < 0.05). In contrast, citrate/creatinine ratios and urinary UA levels were significantly lower in the kidney disease–associated nephrolithiasis group (all *P* < 0.05). No statistically significant differences were observed between the two groups in oxalate/creatinine or phosphate/creatinine ratios (all *P* > 0.05, Table 3).

**Table 3:** Comparison of urinary lithogenic factors between patients with kidney disease–associated nephrolithiasis and those with common nephrolithiasis.

Variable	Kidney disease–associated nephrolithiasis (*n* = 150)	Common nephrolithiasis (*n* = 84)	*t* value	*P* value
Calcium/Creatinine (mmol/mmol)	0.29 ± 0.25	0.21 ± 0.12	2.758	0.006
Oxalate/Creatinine (mmol/mmol)	0.06 ± 0.16	0.04 ± 0.03	1.134	0.258
Phosphate/Creatinine (mmol/mmol)	1.66 ± 0.88	1.57 ± 0.60	0.835	0.405
Citrate/Creatinine (mmol/mmol)	0.12 ± 0.08	0.18 ± 0.12	4.575	< 0.001
Magnesium/Creatinine (mmol/mmol)	0.32 ± 0.24	0.22 ± 0.12	3.574	< 0.001
Urinary UA (mmol/L)	2.11 ± 1.43	4.26 ± 0.98	12.256	< 0.001

#### 3.2.3 Multivariate analysis of factors associated with kidney disease–associated nephrolithiasis

To exclude the potential confounding effects of age, sex, hypertension, diabetes mellitus, and other variables on renal function parameters, and to further clarify the independence of differences observed between patients with kidney disease–associated nephrolithiasis and those with common nephrolithiasis, multivariate logistic regression analysis was performed. Group assignment (kidney disease–associated nephrolithiasis = 1; common nephrolithiasis = 0) was set as the dependent variable, while baseline characteristics, renal function parameters, and urinary lithogenic factors were entered as independent variables after appropriate coding. The multivariate logistic regression model was adjusted for sex, age, BMI, history of smoking, history of alcohol consumption, systolic blood pressure, diastolic blood pressure, hypertension, diabetes mellitus, RBC, WBC, NEU, hemoglobin concentration, FBG, TG, HbA1c, ALT, and AST. The results demonstrated that Scr, BUN, PCR, eGFR, calcium/creatinine ratio, citrate/creatinine ratio, magnesium/creatinine ratio, and urinary UA were independent factors associated with kidney disease–associated nephrolithiasis (all *P* < 0.05, Table 4).

**Table 4:** Multivariate analysis of factors associated with kidney disease–associated nephrolithiasis.

Variable	Assignment	Regression coefficient (β)	Standard error	*P* value	OR	95% CI
Scr	Measured value	0.141	0.040	< 0.001	1.151	1.065–1.244
BUN	Measured value	0.146	0.048	0.002	1.158	1.054–1.272
PCR	Measured value	2.306	0.408	< 0.001	10.033	4.514–22.301
eGFR	Measured value	−0.039	0.020	0.048	0.962	0.926–0.999
Calcium/Creatinine	Measured value	2.406	0.975	0.014	11.087	1.640–74.940
Citrate/Creatinine	Measured value	−7.461	1.746	< 0.001	0.002	0.001–0.018
Magnesium/Creatinine	Measured value	2.889	1.065	0.007	17.983	12.231–24.943
Urinary UA	Measured value	−0.938	0.365	0.010	0.392	0.192–0.800

#### 3.2.4 Correlation analysis between renal function parameters and urinary lithogenic factors in patients with kidney disease–associated nephrolithiasis

Spearman rank correlation analysis demonstrated that, among patients with kidney disease–associated nephrolithiasis, Scr was significantly positively correlated with calcium/creatinine and magnesium/creatinine ratios, and significantly negatively correlated with citrate/creatinine ratio (*r* = 0.884, 0.869, and −0.594, respectively; all *P* < 0.05). BUN was also significantly positively correlated with calcium/creatinine and magnesium/creatinine ratios, and negatively correlated with citrate/creatinine ratio (*r* = 0.565, 0.562, and −0.431, respectively; all *P* < 0.05). Similarly, PCR showed significant positive correlations with calcium/creatinine and magnesium/creatinine ratios, and a significant negative correlation with citrate/creatinine ratio (*r* = 0.806, 0.806, and −0.669, respectively; all *P* < 0.05). In contrast, eGFR was negatively correlated with calcium/creatinine and magnesium/creatinine ratios, and positively correlated with citrate/creatinine ratio (*r* = −0.636, −0.636, and 0.568, respectively; all *P* < 0.05). No significant linear correlations were observed between Scr, BUN, PCR, or eGFR and other urinary components, including oxalate/creatinine ratio, phosphate/creatinine ratio, or urinary UA (all *P* > 0.05).

### 3.3 Analysis of urinary lithogenic factors

#### 3.3.1 Analysis of urinary lithogenic factors in patients with kidney disease–associated nephrolithiasis

After adjustment for sex, age, BMI, smoking history, alcohol consumption history, systolic blood pressure, diastolic blood pressure, hypertension, diabetes mellitus, RBC, WBC, NEU, hemoglobin concentration, FBG, TG, HbA1c, ALT, and AST, further analysis was conducted within subgroups of patients with confirmed nephrolithiasis. In the renal transplant subgroup, the citrate/creatinine ratio was identified as the core urinary lithogenic factor (*P* < 0.05). In the CKD stages 1–2 subgroup, the calcium/creatinine ratio was identified as the core urinary lithogenic factor, while urinary UA was identified as a secondary urinary lithogenic factor (*P* < 0.05). In the CKD stages 3–4 subgroup, citrate/creatinine ratio and urinary UA levels were significantly altered across subtypes (*P* < 0.05, Table 5), suggesting marked metabolic disturbances in this group. Based on the prespecified risk threshold, with hypocitraturia defined as a urinary citrate-to-creatinine ratio < 0.22 mmol/mmol, the prevalence of hypocitraturia in each subgroup was as follows: 94.2% (65/69) in the renal transplant group, 100% (45/45) in the CKD stages 3–4 group, 88.9% (32/36) in the CKD stages 1–2 group, and 66.7% (56/84) in the contemporaneous common nephrolithiasis group. These findings suggest that impaired renal function and post-transplant status are closely associated with a high prevalence of hypocitraturia.

**Table 5:** Analysis of urinary lithogenic factors in subtypes of kidney disease patients with nephrolithiasis.

Variable	Renal Transplant Group (*n* = 69)	CKD Stages 1–2 Group (*n* = 36)	CKD Stages 3–4 Group (*n* = 45)	*Z* value	*P* value
Calcium/Creatinine (mmol/mmol)	0.33 ± 0.31	0.34 ± 0.23	0.20 ± 0.14	4.476	0.013
Oxalate/Creatinine (mmol/mmol)	0.06 ± 0.03	0.05 ± 0.04	0.05 ± 0.04	1.460	0.236
Phosphate/Creatinine (mmol/mmol)	1.83 ± 1.01	1.50 ± 0.72	1.53 ± 0.74	2.421	0.093
Citrate/Creatinine (mmol/mmol)	0.17 ± 0.11	0.15 ± 0.11	0.11 ± 0.09	4.514	0.013
Magnesium/Creatinine (mmol/mmol)	0.36 ± 0.32	0.30 ± 0.15	0.29 ± 0.15	1.363	0.259
Urinary UA (mmol/L)	2.25 ± 1.39	2.56 ± 1.54	1.51 ± 1.10	6.820	0.002

In this study, the classification of “core lithogenic factors” and “secondary lithogenic factors” was based on statistical criteria derived from the strength of association between each urinary parameter and kidney disease-associated nephrolithiasis, compared with the common nephrolithiasis group, in multivariable logistic regression analysis. Specifically, factors with P < 0.05 and the largest absolute regression coefficients, corresponding to odds ratios showing the greatest deviation from 1, were defined as core factors, whereas factors with P < 0.05 but weaker associations were defined as secondary factors. This classification was intended to reflect the relative prominence of each parameter within the study population and represents an observational association rather than a definitive conclusion regarding their primary or secondary pathophysiological roles in stone formation.

## 4 Discussion

Kidney stones are characterized by a high risk of recurrence and complex stone composition, which poses significant challenges for clinical diagnosis and may hinder the effective implementation of subsequent therapeutic strategies (18). Traditional clinical diagnosis has largely relied on physician experience and routine laboratory findings, yet such methods have inherent limitations (19). With the advancement of medical technology, an increasing number of biomarkers and novel detection methods have been introduced into the diagnostic and therapeutic process for kidney stones, thereby laying a solid foundation for precision medicine in clinical practice.

The complexity of kidney stone composition is mainly reflected in the presence of multiple mixed stone components, which are closely associated with metabolic abnormalities. For example, in patients with calcium oxalate stones, common metabolic abnormalities include hypercalciuria, hyperoxaluria, and hypocitraturia (20, 21). Studies have shown that stones with two or more components are often associated with more intricate metabolic disorders (22). For instance, patients with mixed calcium oxalate and UA stones tend to present with hyperoxaluria, hyperuricosuria, and low urinary pH. Currently, research on stone composition has primarily focused on the general kidney stone population, while Renal calculi in patients with kidney disease remain limited. Although reduced urinary citrate excretion accompanying declining renal function is a well-recognized physiological phenomenon in nephrology, previous studies have largely focused on a single type of kidney disease or have not systematically stratified patients according to disease subtype. The principal novelty of this study does not lie in reconfirming the presence of hypocitraturia itself, but rather in systematically comparing, within a single research framework, the urinary lithogenic profiles of patients with nephrolithiasis across three distinct kidney disease subgroups: renal transplant recipients, patients with CKD stages 1–2, and patients with CKD stages 3–4. In addition, this study quantified the subgroup-specific distribution of abnormalities in different lithogenic factors, including the urinary calcium-to-creatinine ratio, urinary citrate-to-creatinine ratio, and urinary uric acid, as well as their distinct patterns of association with renal function indicators. The present study analyzed urinary lithogenic factors in patients with different subtypes of kidney disease complicated by nephrolithiasis. The results demonstrated that, in the renal transplant group, the core urinary lithogenic factor was the citrate/creatinine ratio, whereas in the CKD stages 1–2 group, the calcium/creatinine ratio was identified as the primary lithogenic factor and urinary UA as a secondary lithogenic factor (all *P* < 0.05). In the CKD stages 3–4 group, citrate/creatinine ratio and urinary UA levels were also significantly lower than those in the other subtypes, suggesting the presence of marked metabolic disturbances in this group as well. The identification of these subtype-specific characteristics provides evidence for developing differentiated stone-prevention strategies tailored to specific disease types, representing the principal methodological and clinical contribution of this study relative to the existing literature. In addition, the findings of this study indicate that the metabolic background underlying stone formation differs among kidney disease subtypes. Notably, hypocitraturia appears to be particularly prominent in renal transplant recipients and patients with CKD stages 3–4, and may represent one of the key mechanisms contributing to secondary nephrolithiasis in these populations. As a key risk factor for stone formation, the core pathophysiological mechanism of hypocitraturia is closely associated with citrate metabolism within proximal tubular cells. Metabolic acidosis represents the primary cause of decreased urinary citrate excretion by inducing intracellular acidosis, activating ATP-citrate lyase, promoting citrate degradation, and enhancing its tubular reabsorption.

In addition, hypokalemia, hormonal effects such as angiotensin II, high-protein diets, and certain medications (e.g., thiazide diuretics) also play regulatory roles in this process. Data from prior studies indicate that more than 55% of patients with calcium-based stones exhibit significantly reduced urinary citrate excretion, and even among stone formers without other metabolic abnormalities, up to 48% show low citrate levels in urine (23). Urinary citrate exerts an inhibitory effect on the formation of calcium oxalate stones. It can bind with urinary calcium ions to form highly soluble calcium citrate complexes, thereby reducing the concentration of free calcium ions in urine and decreasing the saturation levels of calcium oxalate and calcium phosphate (24). In addition, citrate can improve urinary pH by enhancing the activity of substances such as pyrophosphate. In patients with kidney disease, pathological conditions including tubular dysfunction, renal tubular acidosis, urinary tract infection, chronic glomerulonephritis, and cystinuria may reduce citrate secretion, thereby leading to hypocitraturia. Meanwhile, factors such as hypokalemia and the acid load associated with a high-protein diet may further decrease citrate excretion, resulting in a reduced citrate/creatinine ratio (25, 26). It is noteworthy that Secondary lithogenic factors also exhibited subtype-specific patterns across different kidney disease categories. In the CKD stages 1–2 group, urinary UA was identified as a secondary lithogenic factor. In contrast, no statistically significant subtype-specific secondary lithogenic factors were identified in the renal transplant group, suggesting that metabolic abnormalities in these populations may be predominantly driven by a single principal lithogenic factor. This phenomenon may be attributable to the distinct pathophysiological mechanisms underlying different kidney disease states. For instance, although increased magnesium excretion in renal transplant recipients did not reach statistical significance in the present study, previous literature has suggested that it may be associated with immunosuppressant-induced renal tubular magnesium wasting. The hypothesis that declining renal function in patients with chronic kidney disease may impair oxalate clearance and thereby induce mild hyperoxaluria is primarily based on previous reports in the literature. However, no significant differences in the urinary oxalate-to-creatinine ratio were observed among the kidney disease subgroups in the present study. Therefore, this mechanism was not directly confirmed in our study population and requires further validation in studies with larger sample sizes.

It is noteworthy that the present study also revealed that urinary UA levels in patients with kidney disease–associated nephrolithiasis were significantly lower than those in patients with common nephrolithiasis, which contrasts with previous clinical findings suggesting that reduced eGFR is typically associated with increased UA retention. This discrepancy may be explained by differences in dietary patterns and metabolic states between the two populations. Specifically, most patients with common nephrolithiasis in this study were from coastal regions, where diets are rich in seafood and other purine-containing foods, potentially leading to increased UA excretion. In contrast, patients with kidney disease often exhibit impaired renal function, reduced appetite, and dietary restrictions (such as low-purine diets), resulting in relatively lower UA levels. Additionally, the results of the present study demonstrated that among patients with different kidney disease subtypes (renal transplantation, CKD stages 1–2, and CKD stages 3–4), the positive rate of hypocitraturia—defined as a citrate/creatinine ratio below the established risk threshold—was relatively high in renal transplant recipients and patients with CKD stages 3–4. Specifically, using a urinary citrate-to-creatinine ratio < 0.22 mmol/mmol as the risk threshold for hypocitraturia, the prevalence of hypocitraturia was 94.2% in the renal transplant group, 100% in the CKD stages 3–4 group, and 88.9% in the CKD stages 1–2 group, all of which were substantially higher than the 66.7% observed in the common nephrolithiasis group. This gradient suggests that declining renal function may directly aggravate hypocitraturia through pathophysiological mechanisms such as metabolic acidosis, while immunosuppressant-related tubular injury may further exacerbate this metabolic abnormality in renal transplant recipients. We hypothesize that renal impairment itself may induce hypocitraturia through a series of pathological alterations—for instance, metabolic acidosis can directly affect tubular cells, enhancing citrate reabsorption and reducing citrate synthesis in the proximal tubules, thereby significantly decreasing urinary citrate excretion. Persistent and severe hypocitraturia represents a key driving factor for rapid stone recurrence and disease progression.

From the perspective of clinical management, identifying the prominent lithogenic profiles determined by statistical associations in patients with different kidney disease subtypes may facilitate the development of more targeted preventive strategies (27). For example, citrate supplementation may be considered for renal transplant recipients in whom hypocitraturia represents the most prominent metabolic abnormality, whereas greater attention should be paid to the regulation of calcium metabolism and dietary intervention in patients with early-stage CKD characterized by hypercalciuria. Previous studies have shown that impaired tubular acidification caused by renal dysfunction may lead to renal tubular acidosis and a reduction in urinary pH, thereby promoting the formation of uric acid and cystine stones. On this basis, the relationship between renal function indicators and nephrolithiasis has increasingly become a focus of clinical investigation (28). In the present study, after carefully controlling for confounding factors such as sex, diet, and comorbidities (e.g., diabetes mellitus and hypertension), Spearman's rank correlation analysis revealed significant linear associations between renal function indicators and certain urinary lithogenic factors (calcium-to-creatinine, magnesium-to-creatinine, and citrate-to-creatinine ratios) among patients with different kidney diseases. These findings suggest a close relationship between renal function changes and stone formation across kidney disease subtypes. As eGFR declines, the kidney's ability to excrete calcium and magnesium diminishes, leading to the formation of calcium–magnesium complexes that interfere with calcium absorption and utilization. This disruption in calcium metabolism and homeostasis may ultimately contribute to hypercalciuria and other metabolic abnormalities.

Notably, subgroup analysis showed that the urinary calcium-to-creatinine ratio in patients with CKD stages 3–4 (0.20 ± 0.14) was significantly lower than that in renal transplant recipients (0.33 ± 0.31) and patients with CKD stages 1–2 (0.34 ± 0.23). This apparently paradoxical finding suggests that urinary calcium excretion does not increase continuously as renal function declines severely, but instead exhibits a dynamic pattern of an initial increase followed by a decrease. Possible mechanisms include the following. In early-stage CKD (stages 1–2), compensatory calcium excretion by residual nephrons may increase. In addition, secondary hyperparathyroidism may promote the release of calcium from bone and reduce tubular calcium reabsorption, thereby increasing urinary calcium excretion. However, as renal function deteriorates further in CKD stages 3–4, the marked decline in eGFR reduces the total amount of calcium filtered by the glomeruli. Meanwhile, reduced production of active vitamin D, decreased intestinal calcium absorption, and phosphate retention with worsening calcium–phosphate imbalance may jointly contribute to a subsequent reduction in urinary calcium excretion (29). Furthermore, although metabolic acidosis, which commonly occurs in advanced CKD, may inhibit tubular calcium reabsorption, this effect may be masked by the substantial reduction in the filtered calcium load. In renal transplant recipients, immunosuppressive agents, such as calcineurin inhibitors, may directly promote renal tubular magnesium and calcium wasting. In addition, although graft function is partially restored, persistent tubular injury may maintain urinary calcium excretion at a relatively high level. This gradient suggests that the relationship between renal function and urinary calcium excretion is not simply linear but is regulated by a complex and dynamic balance involving glomerular filtration, tubular reabsorption, endocrine regulation, and medication-related effects (30). Therefore, when evaluating stone risk in patients at different CKD stages, urinary calcium levels should be interpreted individually according to eGFR stage. Calcium metabolism should be the primary focus in patients with early-stage CKD, whereas in patients with advanced CKD, attention should be paid to the potential tendency toward calcium phosphate stone formation associated with low urinary calcium excretion.

It is essential to acknowledge the various limitations of this study. (1) This study was retrospective in design. Although potential confounding variables were partially controlled through comparative analysis of clinical data, it was not possible to completely eliminate all sources of confounding (e.g., differences in median age, variations in medication use, and heterogeneity in disease stage). In particular, differences in disease activity and treatment regimens (such as the use of diuretics or alkalinizing agents) among patients at different CKD stages and renal transplant recipients may have influenced urinary ion excretion. Although efforts were made to minimize these effects through predefined exclusion criteria, residual confounding cannot be entirely ruled out; (2) The sample population was relatively homogeneous, and the limited sample diversity may affect the generalizability of the results; (3) Multiple testing correction was not performed, which may have increased the risk of Type I error. (4) Other potential confounding factors—such as patient-specific variability, laboratory environment, and temperature—were not considered, which may have affected the accuracy of the study results. (5) Although no statistically differences were observed in baseline characteristics, substantial heterogeneity exists among the subtypes within the kidney disease cohort (renal transplantation, CKD stages 1–2, and CKD stages 3–4) in terms of pathophysiological mechanisms, therapeutic regimens, and disease stages. While combining these subtypes for analysis may facilitate the identification of shared characteristics, it may also obscure subtype-specific differences. (6) The term “kidney disease” encompasses a wide range of disease entities with distinct etiologies and pathophysiological mechanisms. The patients with CKD included in this study predominantly had primary glomerular diseases, such as membranous nephropathy and IgA nephropathy, or secondary glomerular diseases, such as diabetic kidney disease and hypertensive nephropathy. Specific disorders that clearly interfere with stone metabolism, including renal tubular acidosis, were excluded. Nevertheless, even within the same CKD stage, subtle differences in lithogenic profiles may exist among different glomerular disease types. Owing to the limited sample size, this study did not perform detailed subgroup analyses according to specific underlying diseases. Therefore, the current findings are primarily applicable to patients with CKD caused by glomerular diseases and should be generalized cautiously to patients with other etiologies, such as polycystic kidney disease and reflux nephropathy. (7) Although morning urinary pH and serum bicarbonate were routinely measured at enrolment and confirmed to be within the normal ranges in all included participants, and patients with acid–base disorders such as renal tubular acidosis were excluded, acid–base balance plays a central regulatory role in citrate metabolism and stone formation. Single static measurements cannot fully reflect the dynamic 24-h acid–base load or circadian variations in renal acidification capacity. The absence of more detailed dynamic assessment of acid–base status represents a limitation of this study. Future studies should incorporate 24-h urinary metabolic analysis and acid-loading tests to further clarify the role of acid–base status in kidney disease-associated stone formation. (8) Although this study systematically analyzed the associations between urinary lithogenic factors and renal function and inferred the potential metabolic backgrounds of different kidney disease subtypes, no infrared spectroscopy or diffraction analysis data on the actual stone composition were available. Given the substantial differences in the pathophysiological mechanisms of calcium oxalate, calcium phosphate, uric acid, and other stone types, their relationships with renal function indicators and urinary metabolic abnormalities may follow distinct patterns. The absence of stone composition data prevented direct analysis of the correspondence between metabolic abnormalities and stone types and precluded confirmation of whether specific abnormalities in lithogenic factors directly resulted in stones of the corresponding composition in each subgroup. This limitation restricts the depth of interpretation of the findings to some extent. Future prospective studies should therefore incorporate stone composition analysis for further validation.

## 5 Conclusion

In conclusion, compared with stone-forming patients with common nephrolithiasis, those with nephrolithiasis complicated by kidney disease exhibited distinct urinary lithogenic profiles characterized by significantly elevated calcium/creatinine and magnesium/creatinine ratios, along with significantly reduced citrate/creatinine ratios and urinary UA levels. Correlation analysis further demonstrated that renal function parameters (Scr, BUN, PCR, and eGFR) were significantly positively correlated with calcium/creatinine and magnesium/creatinine ratios, and significantly negatively correlated with citrate/creatinine ratio. These findings suggest a close association between declining renal function and abnormalities in the excretion of specific lithogenic factors. Among different kidney disease subtypes, the distribution of urinary lithogenic factors varied. In renal transplant recipients, the citrate/creatinine ratio was identified as the core lithogenic factor, whereas in patients with CKD stages 1–2, the calcium/creatinine ratio served as the primary lithogenic factor and urinary UA as a secondary factor. Collectively, these findings underscore the importance of tailoring metabolic interventions and stone prevention strategies according to the specific lithogenic profiles associated with different kidney disease subtypes. It should be emphasized that the above findings were based on a cross-sectional assessment using a single first-morning urine sample adjusted for urinary creatinine and therefore represent only preliminary screening for metabolic abnormalities and exploratory evidence of associations. The inherent limitations of this method mean that it cannot replace 24-h urine analysis for assessing the overall metabolic burden. Future studies should therefore use the gold-standard method to validate and extend the present findings.

## Data Availability

The original contributions presented in the study are included in the article/supplementary material, further inquiries can be directed to the corresponding author.
